# Rhombic organization of microvilli domains found in a cell model of the human intestine

**DOI:** 10.1371/journal.pone.0189970

**Published:** 2018-01-10

**Authors:** Jonas Franz, Jonas Grünebaum, Marcus Schäfer, Dennis Mulac, Florian Rehfeldt, Klaus Langer, Armin Kramer, Christoph Riethmüller

**Affiliations:** 1 Faculty of Physics, Georg-August-Universität, Göttingen, Germany; 2 Max Planck Institute for Dynamics and Self-Organization, Theoretical Neurophysics, Göttingen, Germany; 3 Institute for Pharmaceutical Technology and Biopharmacy, University of Münster, Münster, Germany; 4 nanoAnalytics GmbH, Centre for Nanotechnology, Münster, Germany; 5 Third Institute of Physics—Biophysics, Georg-August-Universität, Göttingen, Germany; 6 Serend-ip GmbH, Centre for Nanotechnology, Münster, Germany; LAAS-CNRS, FRANCE

## Abstract

Symmetry is rarely found on cellular surfaces. An exception is the brush border of microvilli, which are essential for the proper function of transport epithelia. In a healthy intestine, they appear densely packed as a 2D-hexagonal lattice. For *in vitro* testing of intestinal transport the cell line Caco-2 has been established. As reported by electron microscopy, their microvilli arrange primarily in clusters developing secondly into a 2D-hexagonal lattice. Here, atomic force microscopy (AFM) was employed under aqueous buffer conditions on Caco-2 cells, which were cultivated on permeable filter membranes for optimum differentiation. For analysis, the exact position of each microvillus was detected by computer vision; subsequent Fourier transformation yielded the type of 2D-lattice. It was confirmed, that Caco-2 cells can build a hexagonal lattice of microvilli and form clusters. Moreover, a second type of arrangement was discovered, namely a rhombic lattice, which appeared at sub-maximal densities of microvilli with (29 ± 4) microvilli / μm^2^. Altogether, the findings indicate the existence of a yet undescribed pattern in cellular organization.

## Introduction

Symmetry is an important feature of biology on the molecular, cellular and organ levels. At subcellular organization, the most prominent example are microvilli of enterocytes, which are inevitable for controlled substance uptake. In highly functional transport epithelia, microvilli are densely packed in a so-called brush border, which often is ordered in a hexagonal pattern. Caco-2 cells are a well established *in vitro* model for differentiated enterocytes, whose morphology has mostly been analyzed by electron microscopy [1–3]. These studies described a hexagonal arrangement of microvilli for Caco-2 cells as it has been known from *ex vivo* studies [4, 5]. Up to now, various studies focused on the assembly and composition of the cytoskeleton of single microvilli. Briefly, 19 actin filaments cross-linked by fimbrin and villin form the ultrastructure of a microvillus. These filaments also follow a hexagonal symmetry with a center-to-center spacing of 12 nm [6, 7]. Recently, Crawley et al. detected that intermicrovillar adhesions links are involved in the formation of microvillar cluster, which are among other proteins formed by protocadherins [2, 3].

Pattern formation is well studied in physico-chemistry and hexagonal convection cells were already observed in 1901 by H. Bénard [8]. In 1952, A. M. Turing published a theory on pattern formation of reaction-diffusion systems and linked it to biological morphogenesis [9]. His theory has been expanded to explain a variety of biological phenomena like zebra stripes or the functional organization of the primary visual cortex [10–12]. Ouyang et al. found rhombic patterns in a chlorite-iodide-malonic acid reaction, which also exhibits hexagonal patterns and embedded them in a Ginzburg-Landau theory with broken symmetry [13]. By analogy the question arises, whether microvilli, which can exist in a hexagonal arrangement, would also be able to form a rhombic pattern.

Atomic force microscopy (AFM) extends the methods to investigate the ultrastructure of cells and the morphology and lattice structure of single membrane proteins [14–17]. Furthermore, AFM is able to image surface morphology of cells buffered at physiological pH. Hence, it enables nano-analysis close to physiological state to quantify subcellular nano-structures [18, 19]. This often allows to phenotype peculiar functional states or signaling processes of cultured cells [20–22].

Previous studies demonstrated the ability of AFM to image microvilli. Microvilli were visualized *in vitro* on Madin-Darby canine kidney (MDCK) cells by AFM [23]. Hecht et al. gave an estimate for the number of microvilli on AFM micrographs by determining the overall roughness of alveolar type II cells and revealed comparable results to scanning electron microscopy (SEM) [24]. But none of the studies was able to demonstrate an organization into a regular lattice—potentially because of cultivation on solid, impermeable material. A comparative study of AFM and SEM showed that AFM under semi-dry conditions is able to visualize the brush border in a co-culture of Caco-2 and M-cells. This study demonstrates especially the power of elasticity measurements of the AFM allowing identification of Caco-2 and M-cells while here the focus will be set on the subcellular resolution.

The aim of this study is to examine the ultrastructure by AFM of Caco-2 cells grown on permeable filter supports as used by pharmacological studies. Earlier reports examined samples by both AFM and electron microscopy to combine the specific advantages of each method [25, 26]. Since Caco-2 cells are of human origin, they are frequently used in pharmacological studies for intestinal absorption and they have a high (pre-)clinical impact [27]. Recently, Caco-2 absorption of graphene-oxide was proofed to be highly dependent on their surface morphology [28]. Hence, a good *in vitro* characterization and standardization of the cell material is essential for quality assurance [29–31]. The outstanding importance of the Caco-2 cell line is documented through its approval by the US food and drug administration (FDA) and its use in the Biopharmaceutics Classification System (BCS) [32–34].

Here, we applied AFM and SEM for a more detailed analysis of the subcellular surface organization of Caco-2 cells. The lattice structure of microvilli was detected and evaluated by Fourier analysis and the nano-structures by automated single microvilli detection. In addition to the established hexagonal symmetry of dense microvilli areas, we here found a rhombic arrangement of microvilli, which is yet undescribed in biology. The symmetry type was dependent on the microvillar density.

## Material and methods

### Materials

The stable cell line Caco-2, derived from human colon adenocarcinoma cells, was obtained from Dr. Toni Lindl GmbH (Munich, Germany). Dulbeco’s Modified Eagle Medium (DMEM), MEM amino acids, penicillin/streptomycin, L-alanyl-L-glutamine, phosphate buffered saline (PBS) and FBS superior have been purchased from Biochrom AG (Berlin, Germany). Glutaraldehyde (GDA) 25% was acquired from Sigma-Aldrich Chemie GmbH (Steinheim, Germany).

### Cell culture

Caco-2 cells were cultivated with DMEM, supplemented with 1% (v/v) MEM amino acids, 100 U/mL penicillin, 100 μg/mL streptomycin, 2 mM L-alanyl-L-glutamine and 10% (v/v) FBS superior and stored in a controlled atmosphere with 10% CO_2_ at 37°C. Subcultivation was done three times per week with a ratio of 1:5. Cells of total passage 22–29 and up to passage 7 after thawing were used. For experiments, cells were counted using a Scepter™ 2.0 cell counter (Merck KGaA, Darmstadt, Germany) and 7 x 10^4^ cells/cm^2^ were seeded into 12-well Transwell^®^ clear polyester membrane inserts with a pore size of 3 μm (Corning Incorporated, Tewksbury, USA). For differentiation, cells were cultivated in the inserts for 21 days, while changing medium every three days. Barrier integrity was controlled by measurement of the transepithelial electrical resistance (TEER) for 24 hours, using a cellZscope^®^ (nanoAnalytics, Münster, Germany) **(S4 Fig, Table A in S1 File)**. Afterwards, cells were washed twice with PBS and fixed with a glutaraldehyde 2% (v/v) solution in PBS. Fixed cells were washed again and stored at 4°C for AFM imaging.

### Atomic force microscopy (AFM)

Filter pieces (diameter 8 nm) were cut out of the fixed samples and were imaged in PBS using a Multimode AFM equipped with a Nanoscope III controller and software version 5.30sr3 (Digital Instruments, Santa Barbara, CA, USA) in contact mode at forces below 10 nN. Images were recorded in 90 and 0 degree to the cantilever. Silicon-nitride tips on V-shaped gold-coated cantilevers were used (0.01 N/m, MLCT, VEECO, Mannheim, Germany). Maximum tip speed was 70 μm/s.

Images were recorded with an Asylum Research MFP-3D AFM (closed-loop in xy and z) if AC mode was used. The cantilever with a resonant frequency of 37 kHz (in air) was driven with a frequency of 9.5 kHz in fluid (0.03 N/m, Olympus, Bio-Lever: BL-RC150VB-C1, tip radius 30 nm, gold-coated). The set point was carefully chosen to keep interaction forces at a minimum.

### AFM-Topography analysis

Surface object counting (nAnostic™ method) was performed using proprietary algorithms for AFM-images (Serend-ip GmbH, Munster, Germany) as exemplified in Neuhaus et al. (14). Each nano-object is characterized by individual size (local deviational volume, LDV) and shape. Basically, the experimenter trains an artificial neuronal network with examples of desired structures (machine learning) and then, this routine algorithm is applied to all other AFM-recordings in identical fashion. The border of the microvilli was once calculated using the inflection point and once calculated using the maximum curvature for each individual microvilli. The Fourier-Analysis and the color design was performed by the freeware Gwyddion 2.26 (http://gwyddion.net/). For the Fourier-Analysis a Hann-Window after correction of horizontal stripes and median leveling was applied.

### Scanning electron microscopy (SEM)

The samples were placed upon conductive carbon tape. To enhance the electrical conductivity, the samples were dried and coated with a layer of gold approx. 5 nm thick. The images were taken in standard operation mode with a secondary electron detector by a LEO 1530 VP (Zeiss, Oberkochen, Germany).

### Statistical analysis

Results were considered significant when p < 0.05. Student‘s t-test was performed where appropriate. Presented are the mean values ± s.d. unless otherwise declared.

## Results

### AFM resolves microvilli under aqueous conditions

**Fig 1:**
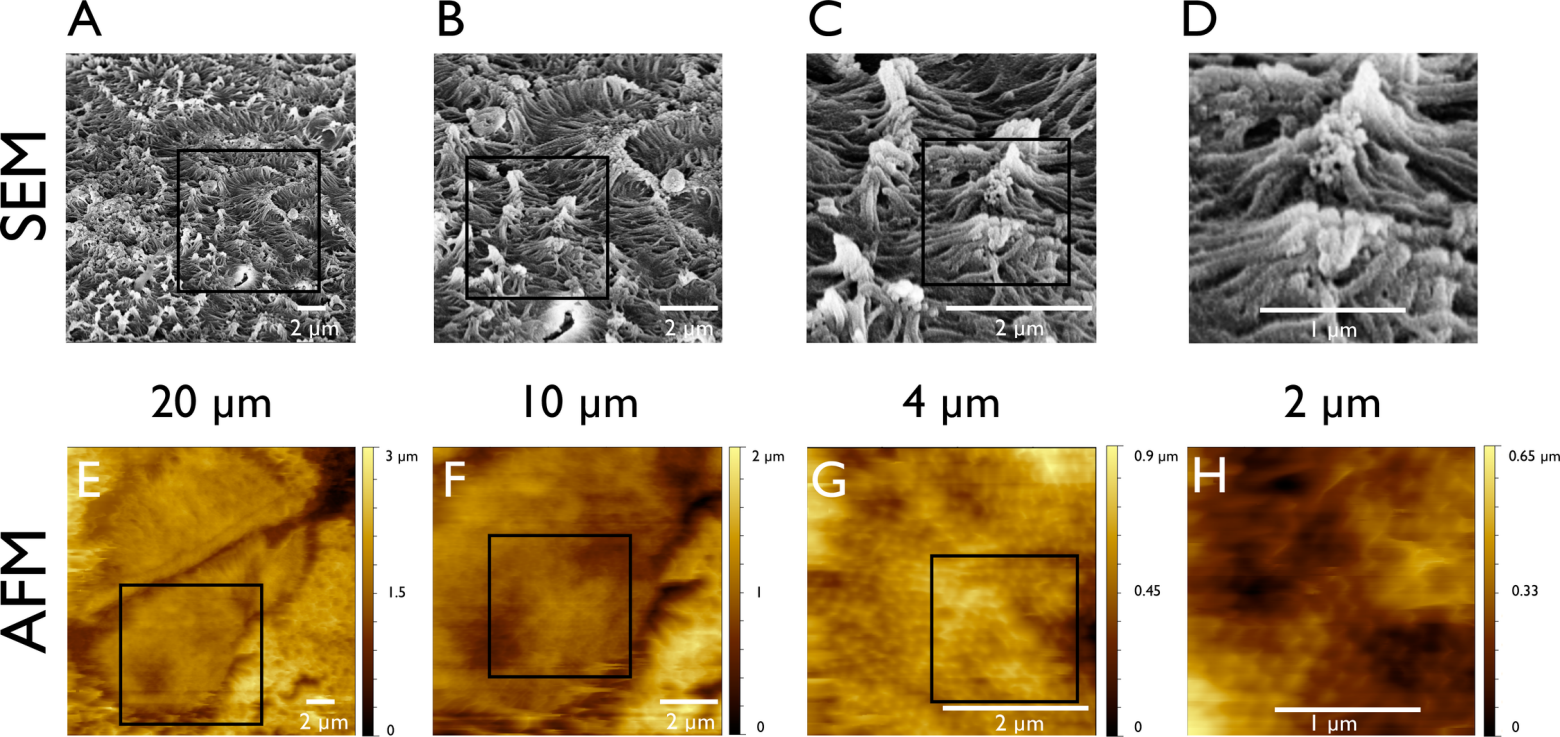
SEM and AFM of Caco-2 cells. (A-D) Scanning electron micrographs of Caco-2 cells at different magnifications. They are compared with atomic force micrographs of Caco-2 cells at the same magnification (E-H). The SEM micrographs display a cell surface completely covered with microvilli (A+B). At higher magnifications the clusters of microvilli appear linked at their tip (C+D). At high magnification the AFM visualizes highly ordered, equally distributed microvilli (G+H).

Atomic force microscopy (AFM) in *contact-mode* and scanning electron microscopy (SEM) was performed to examine the ultrastructure of Caco-2 cells grown *in vitro* on a permeable filter. AFM allows to visualize the nanoscale topography of the cell monolayer in fluid. Here it was found, that Caco-2 cells develop microvilli *in vitro* **(Fig 1A–1H)** and that both methods were able to detect the microvilli. At closer inspection by AFM at high resolution (7nm/px) a regular pattern was visible.

### Caco-2 cells arrange microvilli in an ordered pattern

AFM visualized the arrangement of subcellular ordered patterns of Caco-2 cells. Here, the AFM resolved the lattice structure of microvilli **(Fig 1G and 1H)**. On the SEM images, the microvilli adhered to each other at their tips and built microvillar clusters as previously described by Crawley et al. [2]. A prerequisite for visualizing microvilli and their arrangement was a lateral resolution of better than 15 nm/px. The lattice arrangement of the microvilli was reproducible after repetitive scan of the same area **(S1 Fig)**. Several areas of the Caco-2 cell monolayer displayed cluster like arrangement also in the AFM (**S2 Fig**). Further detailed examination of the lattice structure was performed by Fourier-Analysis.

### Automated quantification of microvilli

**Fig 2:**
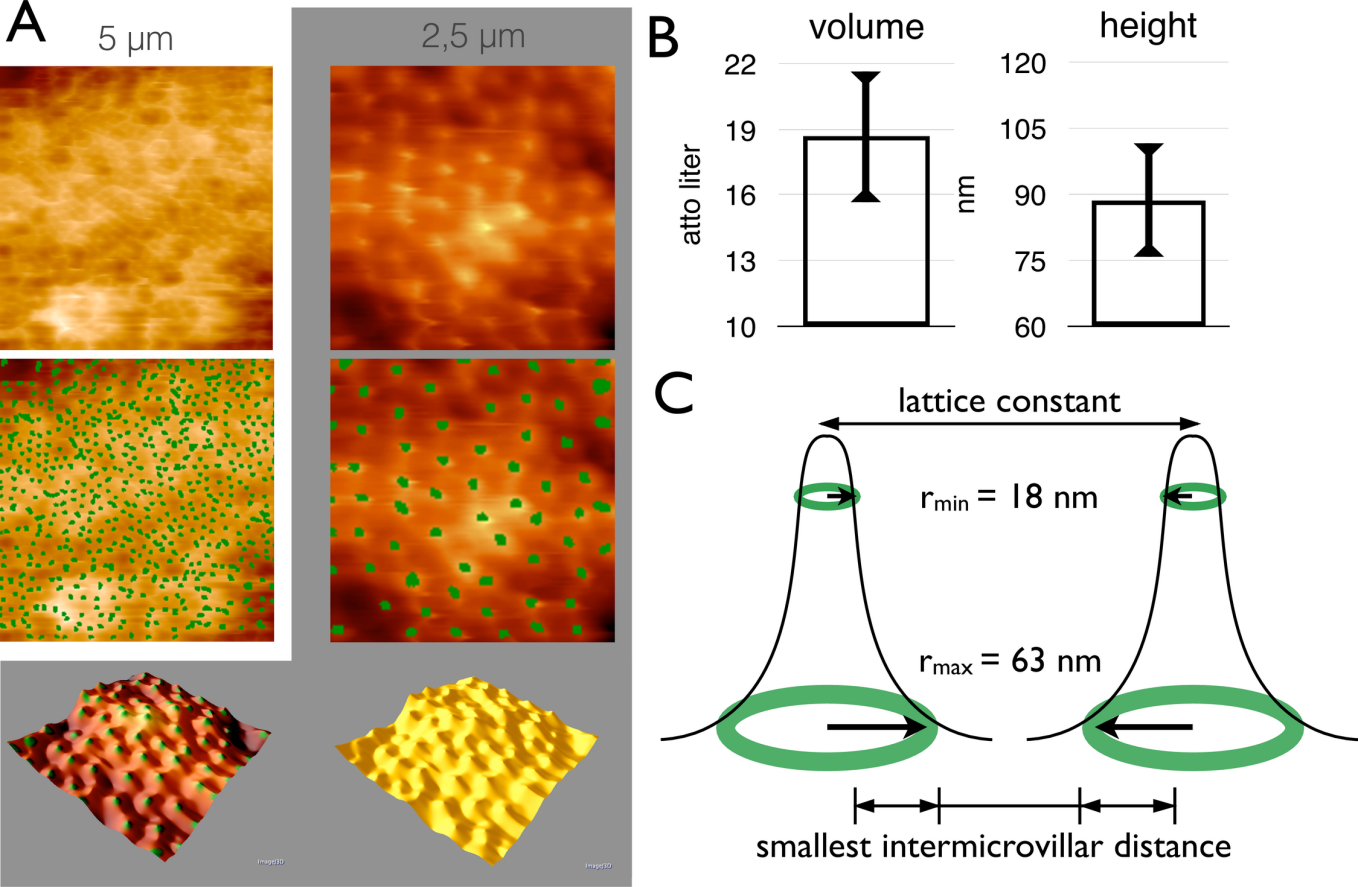
AFM analysis of single microvilli. (A) Atomic force microscopy of Caco-2 cells without and with green labels of the automated detection of microvilli at 5x5 μm^2^ and 2.5x2.5 μm^2^. The last row presents 3D reconstruction of the Caco-2 cell surface at 2.5x2.5 μm^2^ magnification with and without green labelled microvilli. (B) The diagram displays the mean volume ± SD, etc. and the mean height ± SD of one microvillus determined by automated quantification. (C) The minimal and maximal radius calculated by the inflection point and the curvature are presented by green circles in this draft. The SD is represented by the thickness of the circle (r_min_) of 18 ± 2 nm and (r_max_) of 63 ± 5 nm (n = 1574).

In order to analyze the image information objectively, the microvilli locations were quantified by computer vision **(Fig 2A)**. A pattern recognition algorithm was applied, that was specifically designed for AFM-topographies. It evaluates local information of the regions of interest by adopting a trained artificial neural network. This protocol allows for highest inter-rater reliability concerning the dimensions of the microvilli tips **(Fig 2B)** as well as their precise localization and thus their density [microvilli/area].

The microvillar radius cannot be determined directly due to the geometry of the tip of the cantilever (tip convolution). Therefore, a model was created to approximate the radius by setting up an upper and a lower limit for narrowing the expected radius in between. For n = 1574 microvilli the inflection point (second derivative = 0) was calculated and set for the lower limit (r_min_) of 18 ± 2 nm and the maximum of the curvature was taken as the upper limit (r_max_) of 63 ± 5 nm **(Fig 2C, Table B in S1 File)**.

### Fourier analysis of the lattice

**Fig 3:**
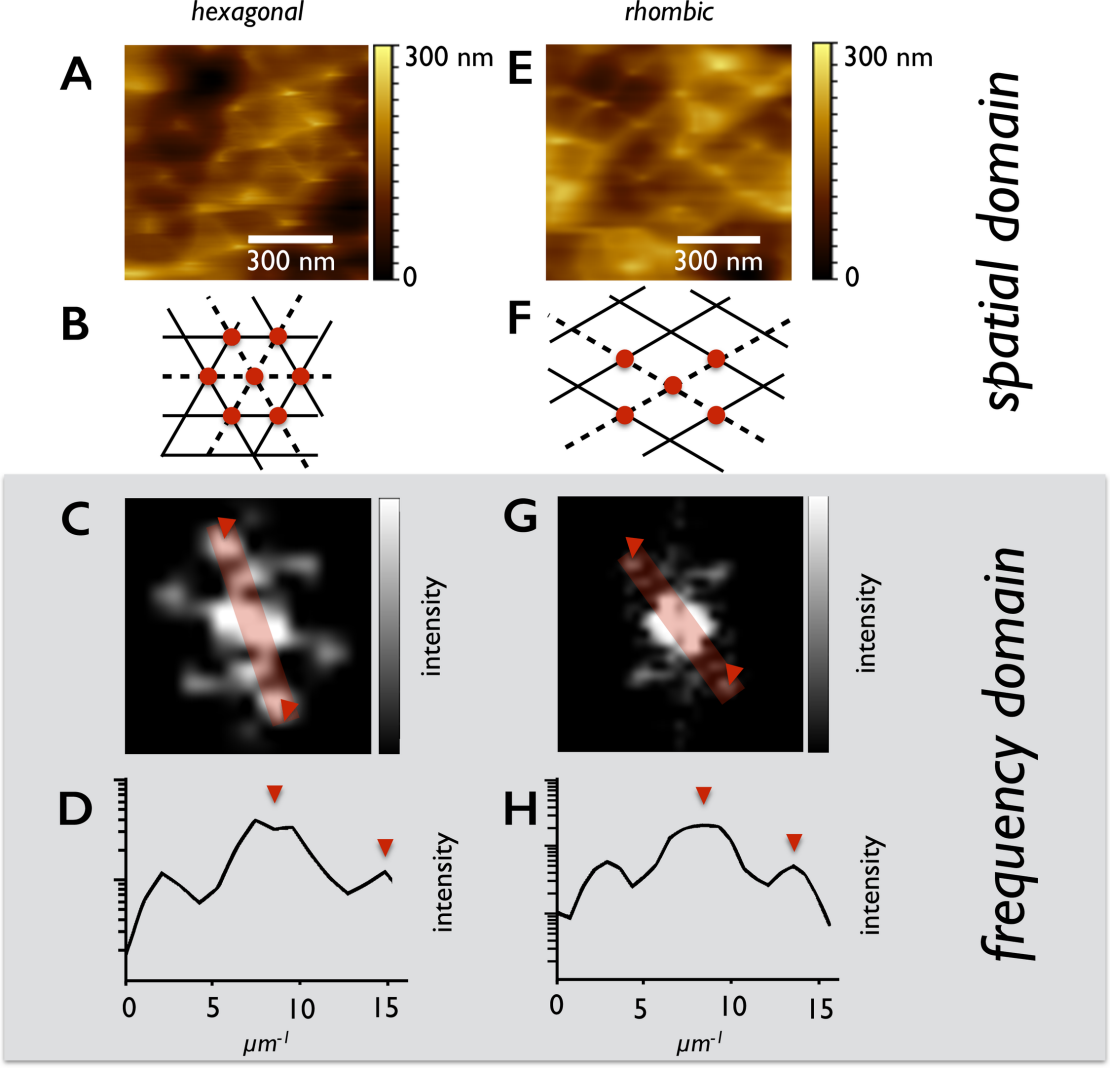
AFM images of different lattice structures of microvilli—Contact mode. The hexagonal (A) and the rhombic (E) lattice structure of the microvilli in the spatial domain imaged by atomic force microscopy. (B) and (F) show the elementary cell of the lattice structure. The red dots represent the microvilli and the lines the lattice planes. (C+G) show the Fourier transformation of (A+E) windowed by the Hann function. The Fourier representations are zoomed into the first Brillouin zones. (D+H) are the plotted profile lines of the red rectangles displayed in the Fourier representations (C+G).

**Fig 4:**
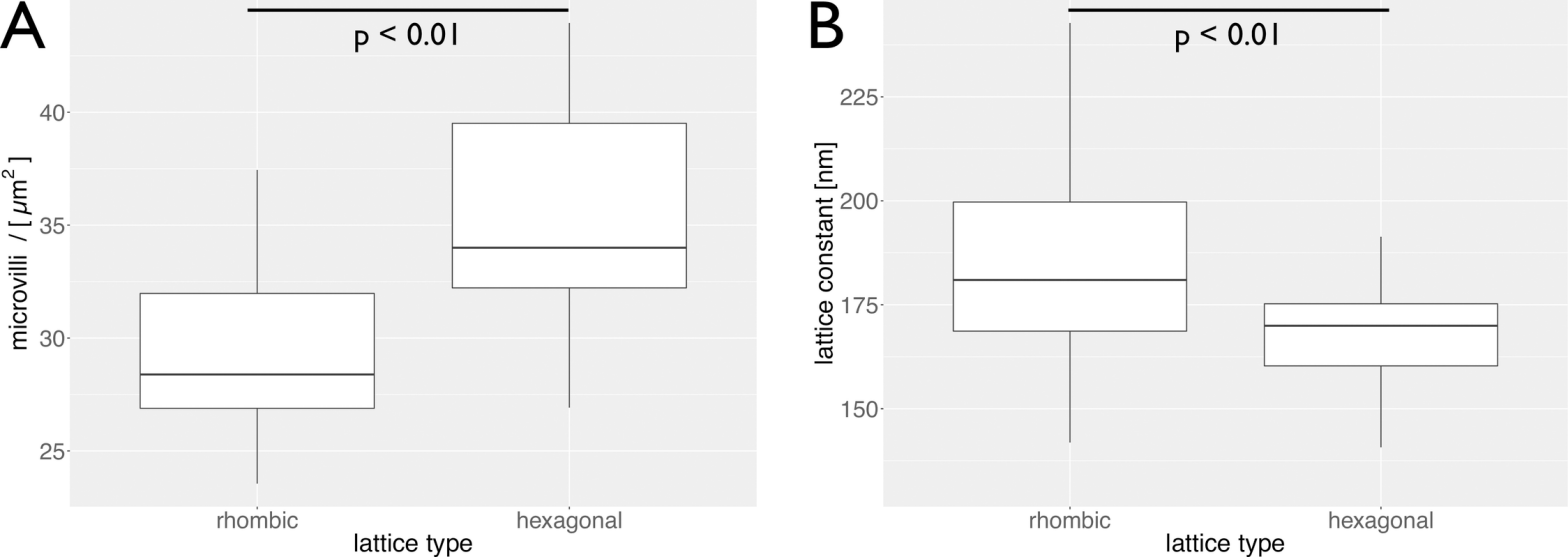
Analysis of different lattice structures of microvilli. (A) Box plot of the median microvillar density for the rhombic and the hexagonal lattice structure. The rectangle represents the 1st and 3rd percentile while the whiskers represent the minimal and maximal measured value. (B) Box plot of the median lattice constant of the rhombic and the hexagonal lattice. The rectangle represents the 1st and 3rd percentile while the whiskers represent the minimal and maximal measured value.

Fourier transformation is a standard tool for the analysis of lattice structures and widely spread i.e. material physics, crystallography, etc. The Fourier transformed image presents the first Brillouin-zone of the lattice structure which is also called the primitive cell. The high-order Brillouin-zones of biological probes are rarely detectable due to their reduced degree of regularity. Analyzing the cellular monolayer hexagonal primitive cells are found **(Fig 3A–3H)**. But the Fourier analysis also revealed another Bravais lattice, the rhombic one. A significant relation could be shown between the microvilli density and the microvillar lattice arrangement by including 37 images of three independent runs with n = 7783 microvilli. At densities of (29 ± 4) microvilli / μm^2^ (1 SD) Caco-2 cells prefer a rhombic lattice. At higher densities from (35 ± 6) microvilli / μm^2^ (1 SD) they arrange in the most dense hexagonal package **(Fig 4A, Table C in S1 File)**.

The lattice constant, which represents the distance between neighboring maxima, is found to be 169 ± 15 nm for the hexagonal packing and 185 ± 24 nm for the rhombic packing **(Fig 4B)**. To evaluate the smallest intermicrovillar spacing, which is important for the structure bearing proteins, both the lattice constant and the diameter were measured.

The smallest intermicrovillar distance was calculated by subtracting the microvillar diameter from the lattice constant. The microvillar diameter (2r) was determined to be in between 36 ± 4 nm and 130 ± 10 nm (see above). This leads to a mean diameter of 80 nm ± 60 nm. The result for the smallest distance was 90 ± 70 nm for the hexagonal and 110 ± 70 nm for the rhombic arrangement (error propagation, only significant digits) **(Fig 2C)**.

### Imaging in AC mode

**Fig 5:**
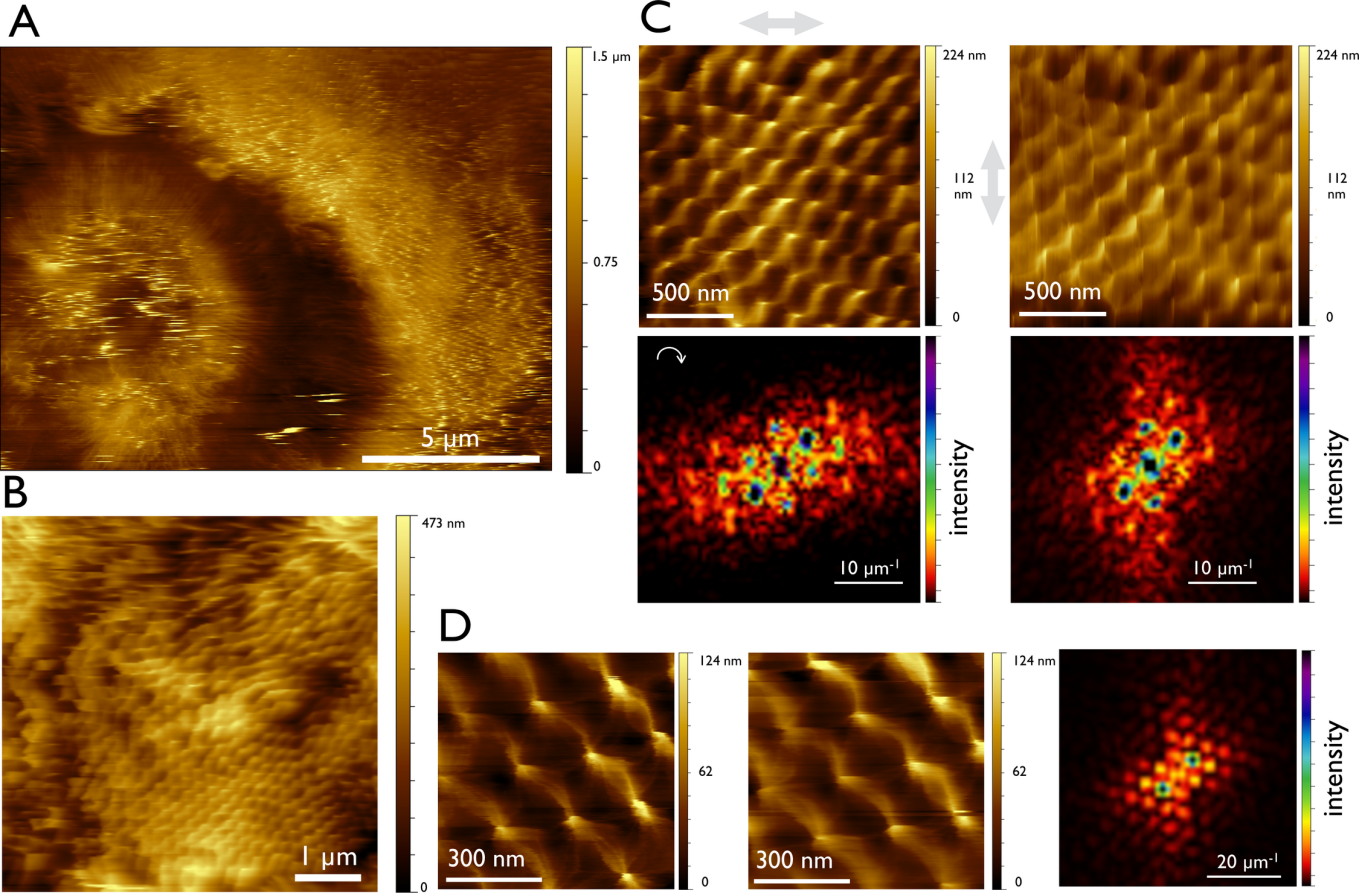
AFM images of the rhombic lattice arrangement of microvilli—AC mode. Series of the same area of Caco-2 cells at different magnifications. (**A**) Overview of Caco-2 cells recorded with 1024 data point per scan line. At this image size the amplitude set point must be decreased to image the whole range in z of 1.5 μm. This leads to adhesion of the tip at some areas. (**B**) This resolution can detect highly and less ordered arrangements of microvilli. (**C**) With a lateral resolution of 6–7 nm/px the lattice arrangement can clearly be identified. Presented are 0 and 90 degree images of the same area (scan direction labelled with light grey arrow) and their Fourier transforms. For better comparison the left Fourier image is rotated as marked by the white arrow. (**D**) At the highest magnification of 2.9 nm/px the intermicrovillar links are visible in detail. A repeated scan is presented to demonstrate image stability. Furthermore the Fourier transform at this level yields to the presentation of harmonic components in the Fourier transformation.

In contrast to the contact mode the cantilever of the AFM is driven with a constant frequency and the amplitude was kept constant. This also called intermittent contact (tapping) mode reduces lateral forces exerted by the probe and helps to exclude that the microvillar arrangement is a result of AFM scanning mode (**Fig 5A–5D**). This advantageously ameliorated the image quality up to a resolution of 2.6 nm/px. The microvilli positions are again invariant upon repetitive scanning and are independent of scan angle 0° or 90°. The lattice was identified as a rhombic one (**Fig 5C**). As a consequence of the increased resolution the Fourier representation displays higher harmonic components, illustrating the deviation of microvillar shape from an ideal sinusoidal curve (**Fig 5D**). The lattice constant obtained in AC mode is 180 nm, which is in good agreement with the value obtained in contact mode.

### Intermicrovillar tip links

At higher resolution (< 7nm/px) we observed straight fibers appearing to connect neighboring microvilli **(Figs 3A and 3E and 5D)**. These intermicrovillar tip links arranged along the symmetry axis of the lattice structure and connected the nearest neighbors. No microvilli were found to be associated with more than six tip links representing the hexagonal lattice. Accordingly, at the rhombic part of the cell surface only four links were detected.

## Discussion

This study focused on the analysis of subcellular patterns. The cell model Caco-2 was chosen due to its high degree of differentiation and physiological function of dense microvilli package, which gained it an indispensable role in pharmaceutical testing setups. AFM was able to reveal various biological 2D-patterns (lattice structures) as exemplified by automated object counting and subsequent Fourier-analysis. First, the commonly known hexagonal packing could be stated but also a second, yet undescribed symmetry in mammalian cells was discovered, namely a rhombic type.

The lattice structure of microvilli is well-known from electron micrographs *ex vivo* [4]. Also *in vitro* Peterson et al. demonstrated by transmission electron microscopy (TEM) that Caco-2 cells develop a growing number of microvilli up to a maximal density [1]. Recently, Crawley et al. observed Caco-2 cells forming clusters of microvilli which turn into a hexagonally arranged brush border [2]. This process is tightly regulated by protocadherin-based intermicrovillar adhesions. Generally, the hexagonal arrangement of microvilli is assumed to be confined to areas with a maximal density (>35 MV/μm) of microvilli. Here, AFM revealed microvillar lattice structures already at lower densities ((29 ± 4) MV/μm), while in SEM the microvilli appear mostly clustered.

Both, the rhombic pattern and the well described observations of microvillar clusters, appear at microvillar densities below the hexagonal packing. The rhombic pattern is observed by atomic force microscopy only, whereas the clusters were also described by different methods like SEM or super-resolution light microscopy. The clusters of microvilli are also detectable by AFM (**S2 Fig**).

Considering the physiological implications of a regular pattern of microvilli below the maximal density of a hexagonal arrangement, several aspects might apply. On the one hand, microvilli are indicative for the state of differentiation: only a well developed brush border warrants full intestinal function. According to the microvillar densities found here and in the light of insights from pattern forming processes, the rhombic lattice is likely to represent an intermediate state of differentiation between the clustered and the hexagonal packing which is potentially realized along the crypt-villus axis. This submaximally differentiated rhombic state might indicate a state of dedifferentiation which is characteristic for adenocarcinoma derived *in vitro* cells. Future studies may unravel, to which extent the respective area fraction of three coexisting patterns varies along with the functional state of a cell barrier. On the other hand, microvilli are central to transport, as active membrane proteins like sucrase are located at the microvilli [35–38]. Generally speaking, the different ordering patterns might influence the working conditions in the unstirred layer and hence the overall efficiency of substance transport (**S3 Fig**).

Apart from the qualitatively new symmetry regime, the dimension of the hexagonal unit cell differs depending on the method used for investigation. For the hexagonal packing, Ferrary et al. determined a lattice constant of about 100–120 nm, which is different to our findings [39]. Here in our study, the lattice constants of the rhombic pattern (184 ± 24 nm) and the hexagonal one (169 ± 15 nm) as measured by AFM were larger than those reported from EM studies (i.e., TEM or quick freeze deep etch EM). Depending on the respective Bravais lattice, the difference measures up to a considerable 90 nm, resembling almost a factor of two.

There are several reasons which might explain the difference in the lattice constant.

First, it is known that the structure building cytoskeletal proteins can vary in their length.

At the basal level of the microvilli the non-erythrocyte spectrins form an organizing network. According to their low force constant, the length of spectrins can vary from 48 to 160 nm, thereby facilitating a rhombic organization [5, 40–42].

Secondly, as reported recently, the trans-heterophilic complex of protocadherin-24 (PCDH24) and mucin-like protocadherin (MLPCDH) is linking the tips of microvilli to form microvillar clusters [2, 3]. Conceivably, these protocadherins might also be involved in generating the rhombic pattern as intermicrovillar tip links are well recognizable in AFM-recordings **(Fig 3A and 3E)**. Here, an alternative splicing and post translational glycosylation might cause variations in intermicrovillar distances [43–45].

Finally, the sample preparation for electron microscopy leads to a shrinkage of the cells [20, 46, 47], although this effect is minimized by quick freeze deep etch EM as used by Crawley et al. [3]. As the measurements by AFM are performed in fluid with no more processing but GDA fixation, they should well approximate the physiological state.

Taken together, microvilli are arranged in 2D-lattices also *in vitro*. This fact could be observed here by applying AFM in fluid to cells grown on permeable filter membranes. Fourier analysis proved both hexagonal and rhombic regions to coexist. The formation of microvillar clusters could also be confirmed by AFM. Overall, the lattice constants under fluid conditions are considerably larger (60-90nm) than reported from EM.

## Conclusion

Caco-2 cells grown on filter membranes are a valuable *in vitro* model for the intestinal barrier—presumably associated with the high degree of surface order. The existence of rhombic symmetry is unprecedented in cells and may add a new facet to the picture of epithelial cell organization—awaiting approval in vivo.

## Supporting information

S1 Fig Repetitive scans. (**A**+**B**) Consecutive images of the same area by AFM (2,5 x 2,5 μm^2^) from repetitive scans in contact mode. Beside a few scan artifacts like the scan line in the upper third the lattice structure of the microvilli is preserved and not influenced by the AFM.
(PDF)

S2 Fig Clustered microvilli. (**A**) SEM image of Caco-2 cells, which demonstrates the parallel existence of ordered microvilli and clusters of microvilli. The microvilli are inclined to side and have a combed like appearance. (**B**) The AFM image of Caco-2 cells displays the heterogeneity of the cells and like (**A**) the clustered appearance parallel to ordered pattern.
(PDF)

S3 Fig Schematic illustration of microvillar arrangement. Shown are the three different types of packing. While the closest packing, the hexagonal arrangement, appears at high densities, the clusters and the rhombic arrangement arise at the same, low density parallel to each other. One difference highlighted here is the increased functional surface of the rhombic packing due to the wider, regular intermicrovillar space.
(PDF)

S4 Fig TEER of Caco-2 cells. The mean TEER over three independent runs with n = [3,2,3] wells for 24 h with error bars of 1 SD.
(PDF)

S1 File **(Table A) Raw data of TEER measurement displayed in S4 Fig**. Three independent runs with [2, 3, 3] wells per run. Measured TEER in [Ω cm^2^] and time in [h]. **(Table B) Raw data of microvilli radius measurement displayed in Fig 2C.** Mean radius of inflection point and maximum curvature for n = 1574 microvilli. **(Table C) Raw data of microvillar lattice measurements in the Fourier transformed image representation displayed in Fig 4.** Images were aligned by the median of each line, leveled by linear planet and corrected for small scars of 4 px maximum width and minimum length of 16 px. The discretized Fast Fourier transformation was performed after windowing the image in the spatial domain by a Hann window. The prominent peaks of the lattice vectors were identified automatically within a manually drawn profile line with sub pixel resolution. The results are provided in a cvs file in columns: Lattice type, Lattice Constant [μm], Image ID, Counts / ROI [μm^-2^].
(ZIP)
