# Supplementary figures and images for: Rhombic organization of microvilli domains found in a cell model of the human intestine
Source: PLoS One. 2018 Jan 10;13(1):e0189970. doi: 10.1371/journal.pone.0189970 (PMC5761853; doi:10.1371/journal.pone.0189970)

A

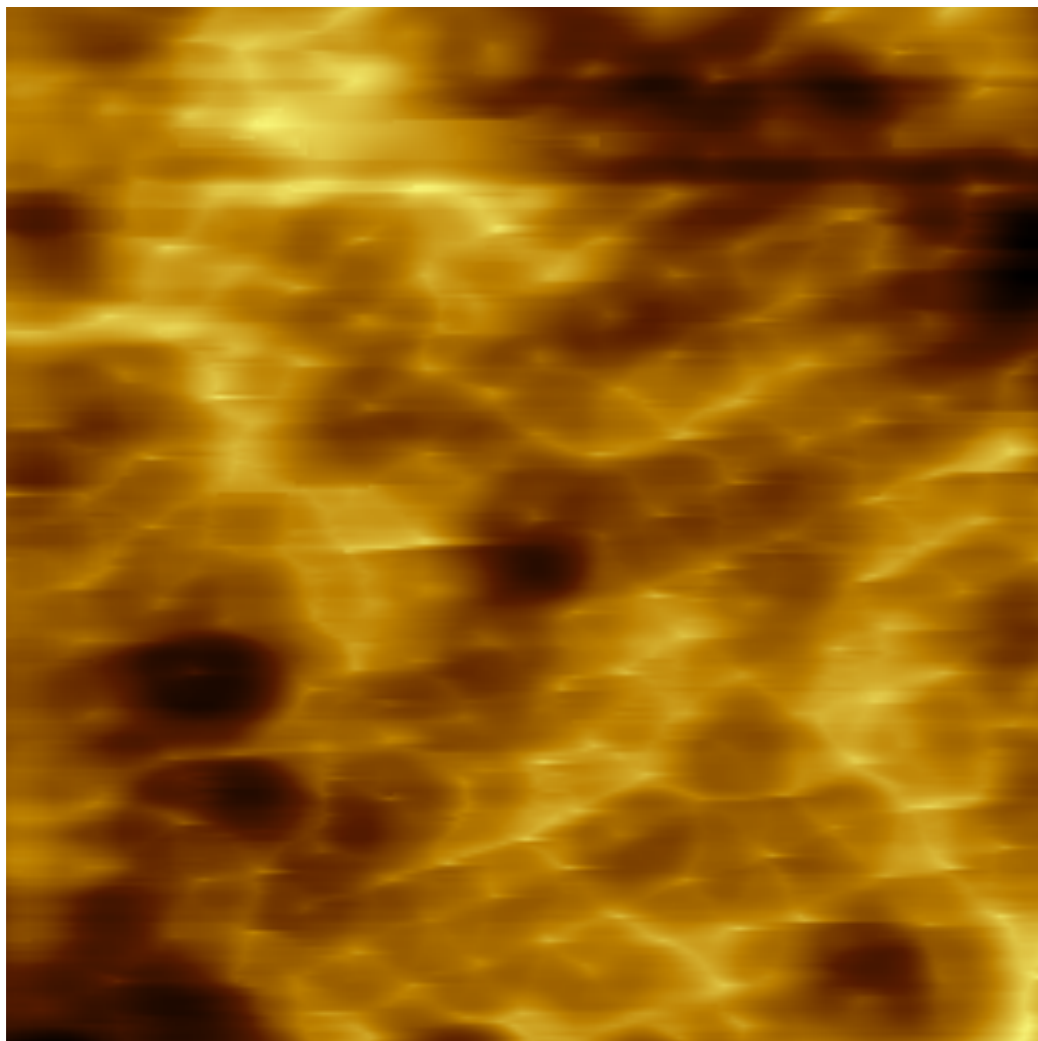

B

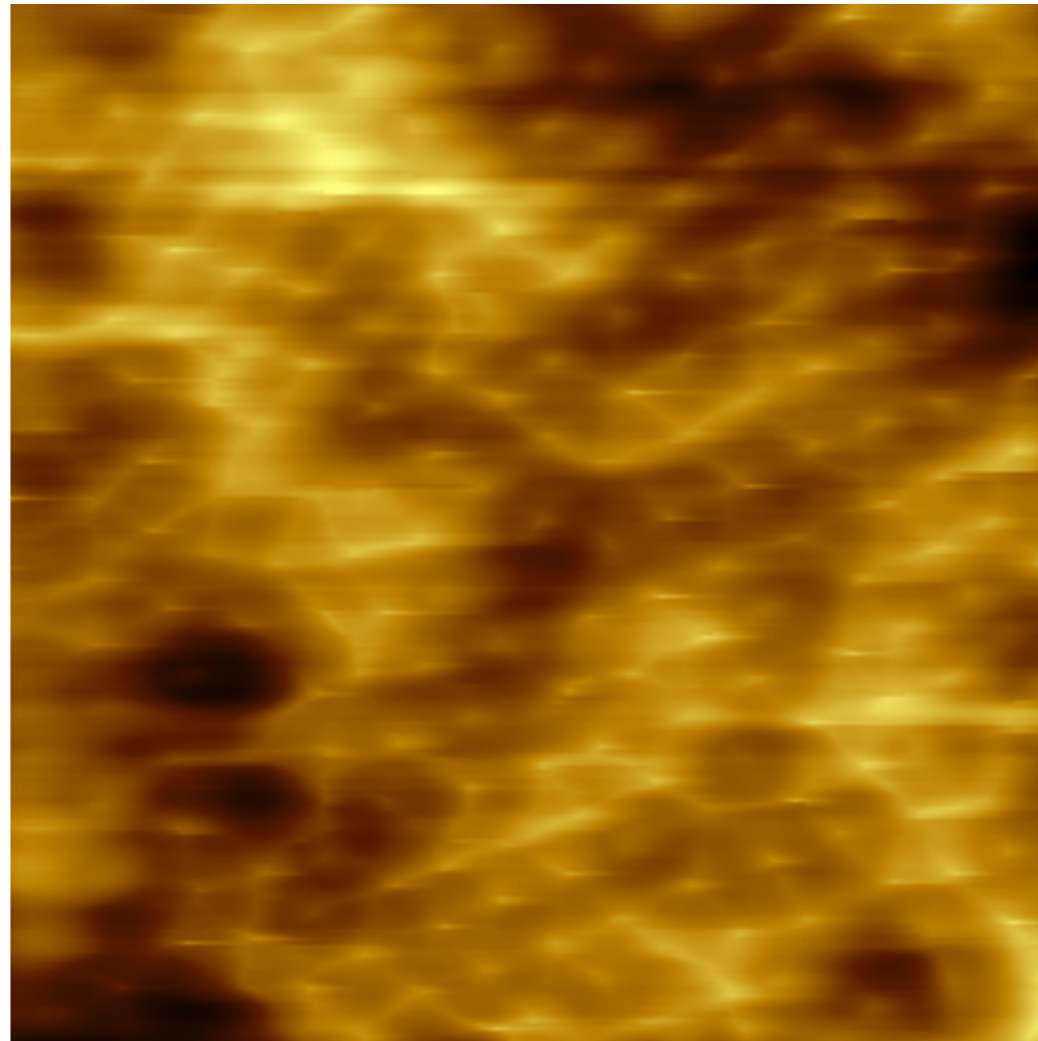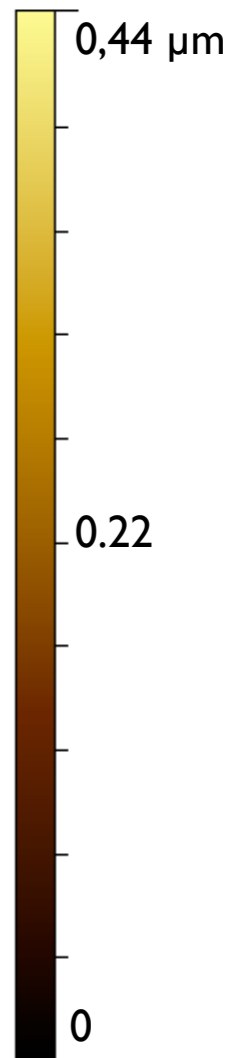

Supplement: S1 Fig — (A+B) Consecutive images of the same area by AFM (2,5 x 2,5 μm2) from repetitive scans in contact mode. Beside a few scan artifacts like the scan line in the upper third the lattice structure of the microvilli is preserved and not influenced by the AFM. (PDF) [file pone.0189970.s001.pdf]

A

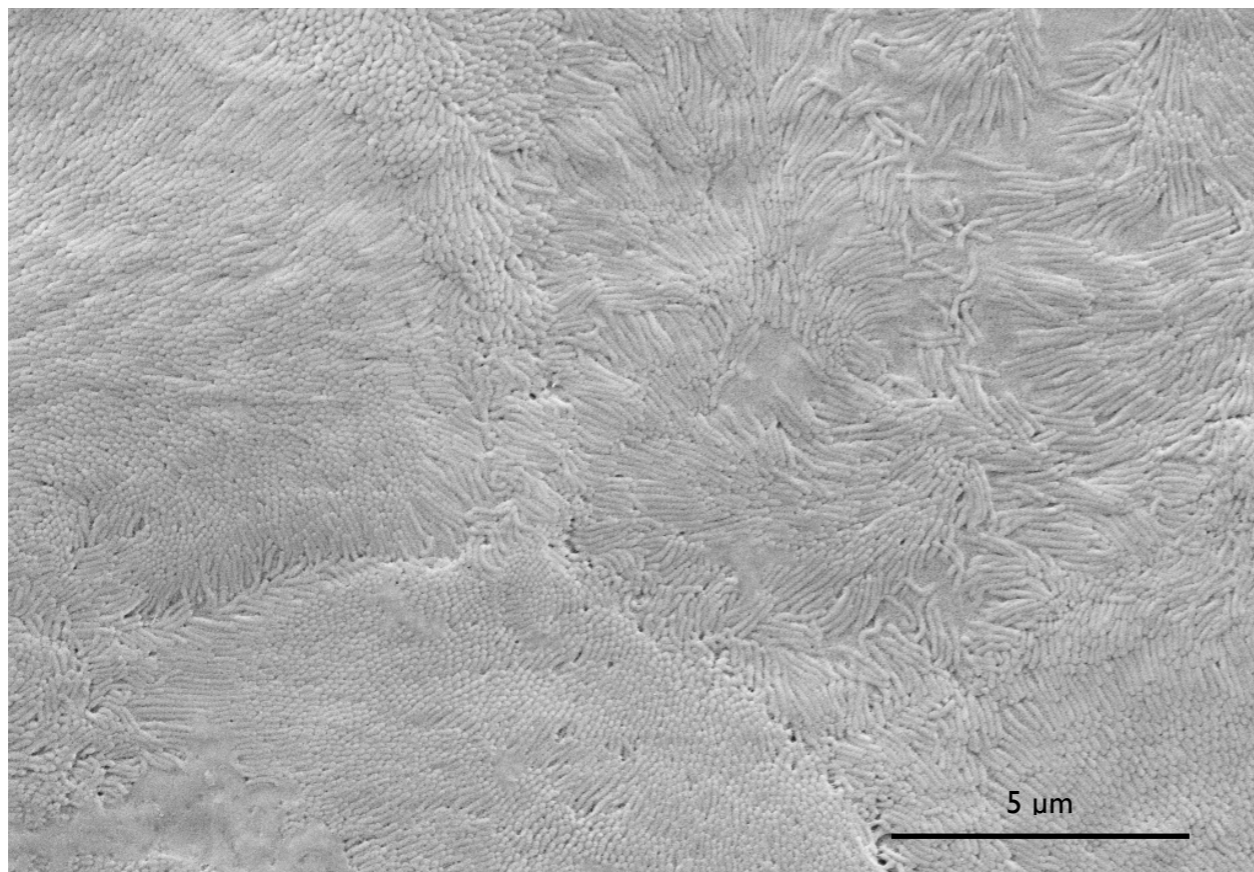

B

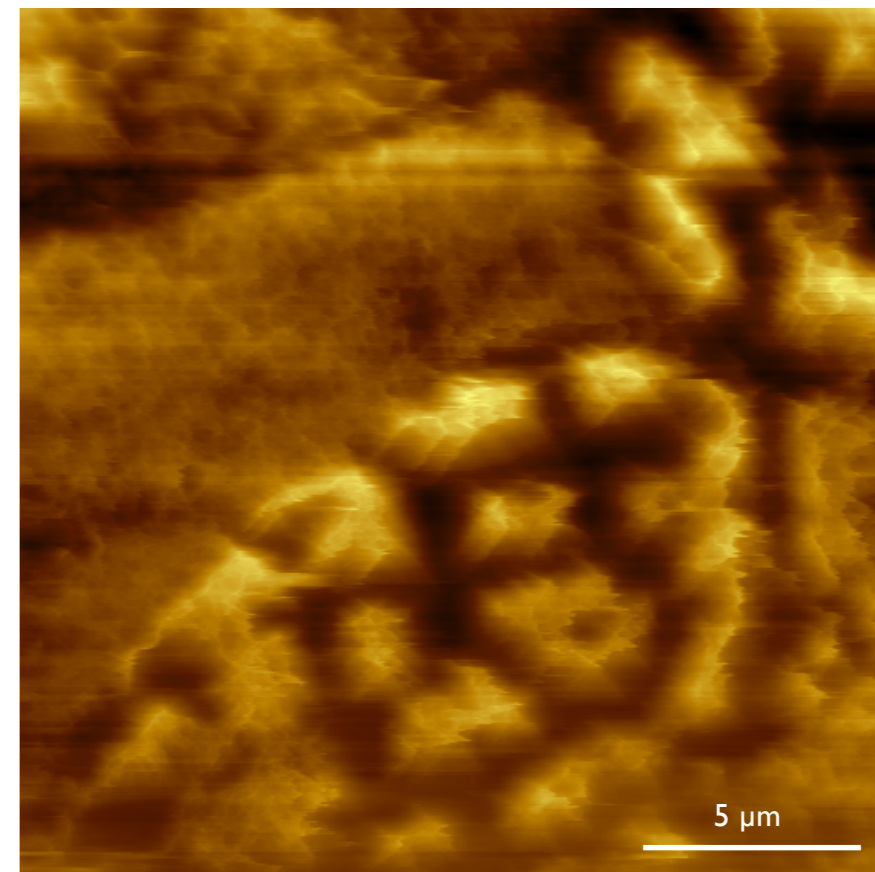

Supplement: S2 Fig — (A) SEM image of Caco-2 cells, which demonstrates the parallel existence of ordered microvilli and clusters of microvilli. The microvilli are inclined to side and have a combed like appearance. (B) The AFM image of Caco-2 cells displays the heterogeneity of the cells and like (A) the clustered appearance parallel to ordered pattern. (PDF) [file pone.0189970.s002.pdf]

functional surface

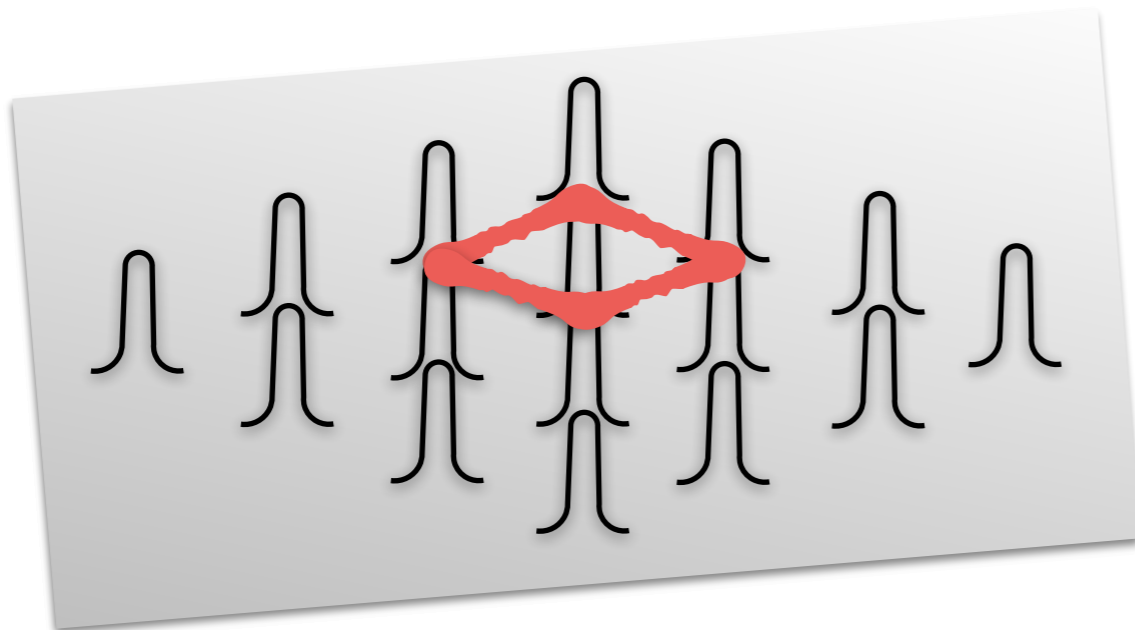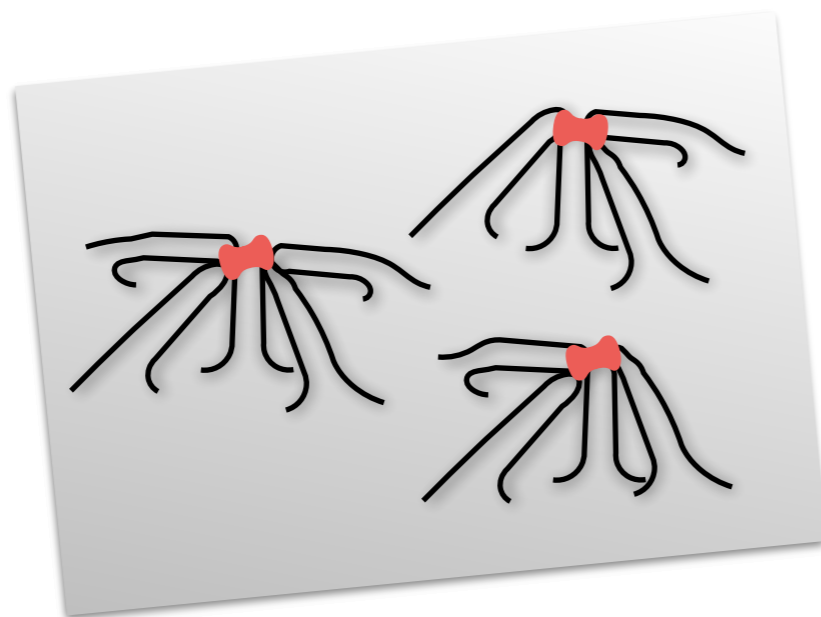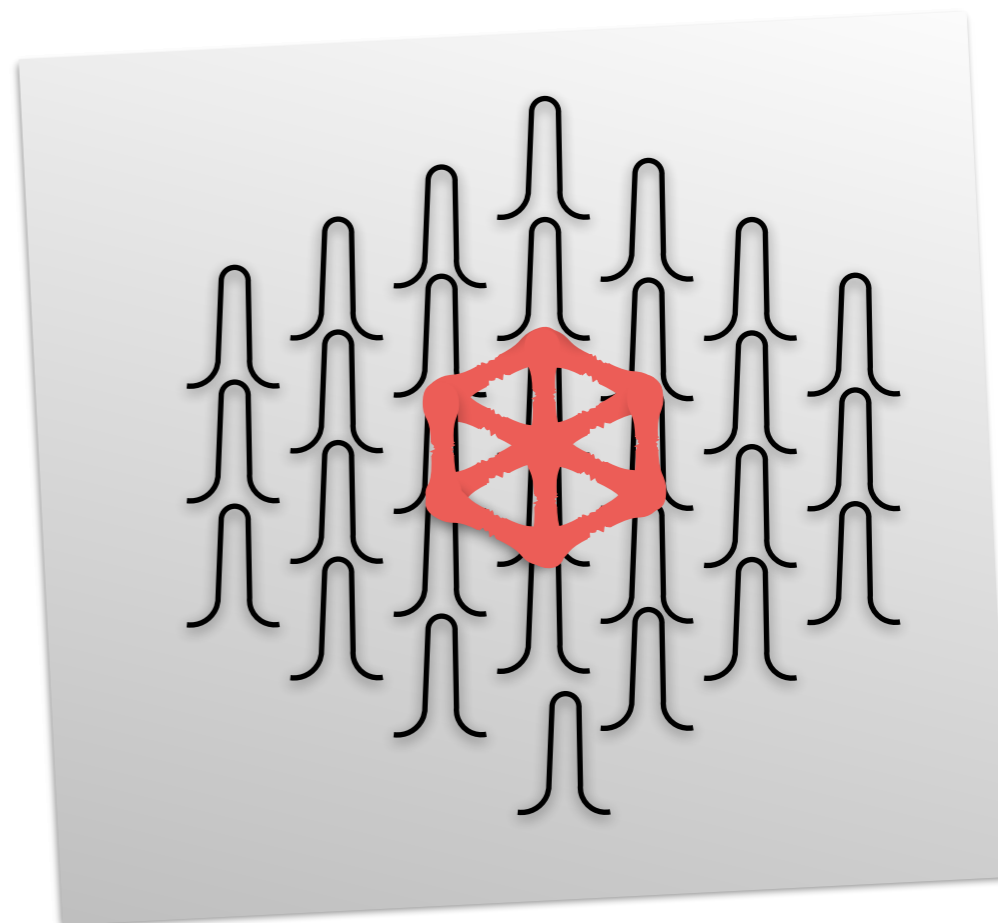

density

Supplement: S3 Fig — Shown are the three different types of packing. While the closest packing, the hexagonal arrangement, appears at high densities, the clusters and the rhombic arrangement arise at the same, low density parallel to each other. One difference highlighted here is the increased functional surface of the rhombic packing due to the wider, regular intermicrovillar space. (PDF) [file pone.0189970.s003.pdf]

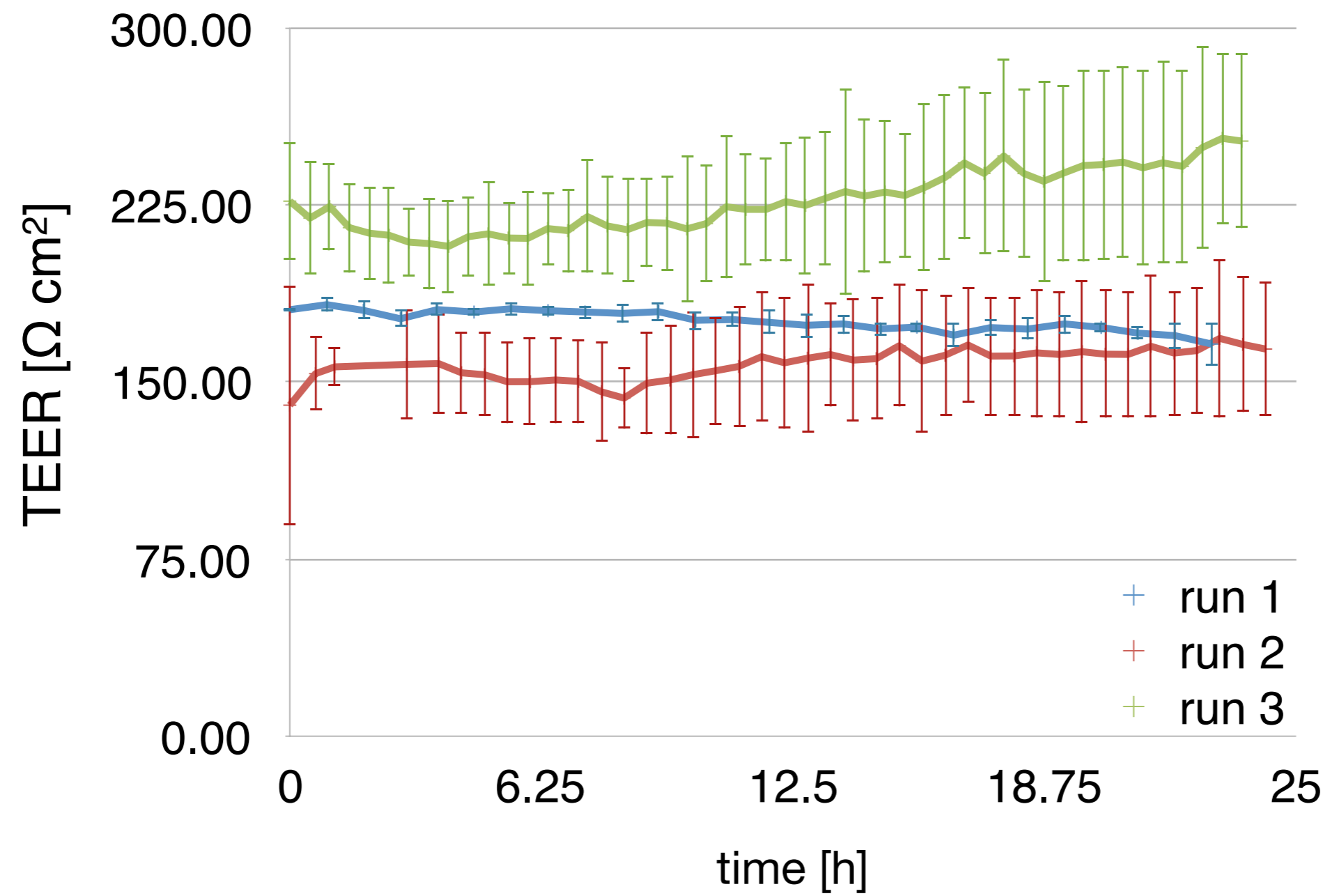

Supplement: S4 Fig — The mean TEER over three independent runs with n = [3,2,3] wells for 24 h with error bars of 1 SD. (PDF) [file pone.0189970.s004.pdf]
